# The Spatiotemporal Patterns and Environmental Effects of Pelagic Communities in the Northwest Pacific Ocean Based on Sound Scattering Layer Descriptors

**DOI:** 10.1002/ece3.73947

**Published:** 2026-07-12

**Authors:** Minghua Xue, Jianfeng Tong

**Affiliations:** ^1^ College of Marine Living Resource Sciences and Management Shanghai Ocean University Shanghai China; ^2^ National Engineering Research Center for Oceanic Fisheries Shanghai China; ^3^ Key Laboratory of Sustainable Exploitation of Oceanic Fisheries Resources Ministry of Education Shanghai China

**Keywords:** deep scattering layer, generalized additive model, pelagic communities, sound scattering layer, the Northwest Pacific Ocean

## Abstract

The sound scattering layer (SSL) plays an important role in maintaining the structure and functioning of marine ecosystems. The spatiotemporal distribution of the SSL largely reflects the distribution of pelagic communities within the water column. Although acoustic data are widely used to investigate the distribution of communities within SSLs, a systematic framework for analyzing underlying environmental mechanisms remains limited. To address this, this study developed a model using SSL descriptors. SSL descriptors provide a new perspective for investigating community distributions in large marine ecosystems by capturing spatiotemporal variability in the structure and functioning of pelagic communities. We characterized the SSL using a suite of SSL descriptors. Descriptors indicative of community biomass then were used to fit generalized additive models. We applied this approach to an acoustic dataset collected in the Northwest Pacific Ocean during 2022–2024 to assess pelagic community distribution patterns and the key environmental drivers. This SSL descriptor‐based modeling framework provides an innovative and robust approach for analyzing relationships between marine environmental variables and pelagic community distributions. Our results indicated that SSL descriptors are effective for monitoring and analyzing large‐scale aggregations of pelagic communities. They support standardized monitoring of pelagic ecosystems and have high practical value in regions where species identification is difficult due to a lack of net sampling. This study provides a reference for further applications of SSL descriptors.

## Graphical abstract

This study developed generalized additive models using SSL descriptors, providing an innovative approach for analyzing relationships between marine environmental variables and pelagic community distributions.

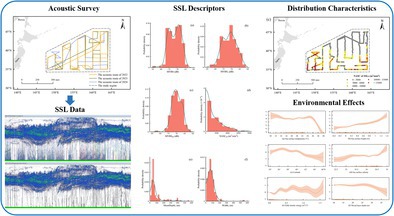

## 1 Introduction

The sound scattering layer (SSL) is aggregation bands that are widespread throughout the global ocean (Kim et al. 2022). It is typically formed by dense concentrations of pelagic communities (Blanluet et al. 2019). The SSL includes a wide range of zooplankton, small cephalopods, small fishes, and fish larvae. This banded structure is relatively narrow in the vertical dimension, whereas it can extend for tens to thousands of kilometers horizontally (Simmonds and MacLennan 2008). The SSL contains some of the largest fish resources in the global ocean that remain among the least exploited (Martin et al. 2020).

The SSL is of major ecological importance in maintaining the structure and functioning of marine ecosystems. Communities within the SSL form complex, multi‐trophic food webs and niche structures through diverse interspecific interactions, resulting in ecological networks with tightly coupled structure and closely linked functions. These networks play a central role in stabilizing ecosystem structure, facilitating energy transfer, and sustaining biodiversity. They also represent a key component of midwater ecosystem functioning (Duxbury et al. 2000). Communities within the SSL constitute important prey for larger fishes and marine mammals (Lebourges‐Dhaussy et al. 2000). These diverse taxa co‐occur spatially within the SSL and exhibit pronounced temporal rhythms in activity. They display collective behaviors such as diel vertical migration (DVM). This behavior can alter phytoplankton biomass and community composition in the surface layer (Burkill et al. 1987). It also links trophic structure across depths through repeated vertical movements.

The horizontal distribution of the SSL is linked to the mechanisms underlying biological aggregation and to spatial heterogeneity in the marine environment. It is sensitive to spatial and environmental variability (Hays et al. 2005) and exhibits corresponding spatiotemporal changes. Spatiotemporal variability in the SSL essentially reflects the distribution patterns of communities within the layer. The SSL can therefore serve as an indicator structure in marine ecosystems (Remond 2015). Investigating the spatial distribution of the SSL can improve our understanding of the distribution patterns of pelagic communities and their environmental drivers. It can also provide key evidence for identifying fishing grounds and informing fishing decisions. Clarifying the links between environmental factors and the SSL can help reveal the mechanisms underlying SSL formation and dynamics. This knowledge can support ecosystem‐based fisheries management and marine ecosystem conservation. Previous studies have linked SSL with environmental variables using different methods. Diogoul et al. (2020) related SSL characteristics, including thickness, depth, and acoustic density, to pelagic habitat variables using multivariate analyses and ANCOVA. Receveur et al. (2020) classified acoustic vertical profiles into homogeneous clusters and used XGBoost to relate these clusters to environmental variables and predict vertical echograms in unsampled areas. David et al. (2025) classified acoustic data into echo‐groups using hierarchical clustering, supported their biological interpretation with sampling information, and used XGBoost to relate echo‐group distributions to environmental variables. These studies indicate that the relationship between the SSL and environmental variability can be examined using different modeling frameworks.

Responses of the SSL to environmental variability can be characterized by a suite of SSL descriptors (Mouget et al. 2022). These descriptors cover variables that characterize SSL properties, including the number of SSLs (Urmy et al. 2012), SSL depth (Mouget et al. 2022, 2025), and SSL scattering strength (MacLennan et al. 2002). The SSL can be characterized in terms of geometric structure, scattering strength, and behavioral features by using different descriptors. These descriptors can then be used to infer spatial arrangement, distribution, and coherence of communities within the layer (Proud et al. 2015). They also provide an integrated indication of pelagic ecosystem conditions (Mouget et al. 2022). In addition, SSL structures formed by diverse communities with distinct spatial distributions can be differentiated using SSL descriptors, enabling classification of regional pelagic communities (Longhurst 1998; Proud et al. 2015). Different descriptors have been applied in previous studies, either individually or in limited combinations, to investigate marine ecosystems. Proud et al. (2015) combined multiple descriptors and made important progress towards standardization. Building on this work, Mouget et al. (2022, 2025) further expanded and integrated more than ten descriptors, demonstrating their potential for monitoring large marine ecosystems. Appropriate selection and combined use of descriptors enable multidimensional analyses of SSL structure and ecological functioning and provide important insights into the spatiotemporal distribution of pelagic communities (Mouget et al. 2022). It can also provide a robust acoustic basis for subsequent habitat modeling, resource assessment, and analyses of ecosystem responses.

The North Pacific Ocean was the first oceanic region in which the SSL was discovered (M. W. Johnson 1948). In the Kuroshio‐Oyashio confluence region and its extension, exceptionally complex water mass structure and frontal systems promote the aggregation of biotic communities (Zhu et al. 2024). These conditions provide a favorable ecological basis for SSL formation and development. However, long‐standing constraints related to high survey costs and limited observing platforms have left the spatiotemporal characteristics of the SSL in this region poorly understood, which constrains a deeper understanding of the regional marine ecosystem and pelagic communities.

This study analyzed data from integrated fisheries resource surveys conducted in the Northwest Pacific Ocean during 2022–2024 and analyzed SSL characteristics using multiple SSL descriptors. At an appropriate spatial scale, descriptors indicative of community biomass were used to fit a generalized additive model (GAM). The main objectives of this study were: (1) to characterize the spatiotemporal distribution of pelagic communities in this region; (2) to use GAM to examine relationships between the acoustic density of pelagic communities and marine environmental factors. This SSL descriptor‐based modeling framework provides an innovative approach and offers robust support for analyzing relationships between marine environmental variables and community distributions.

## 2 Materials and Methods

### 2.1 Survey Information

**FIGURE 1:**
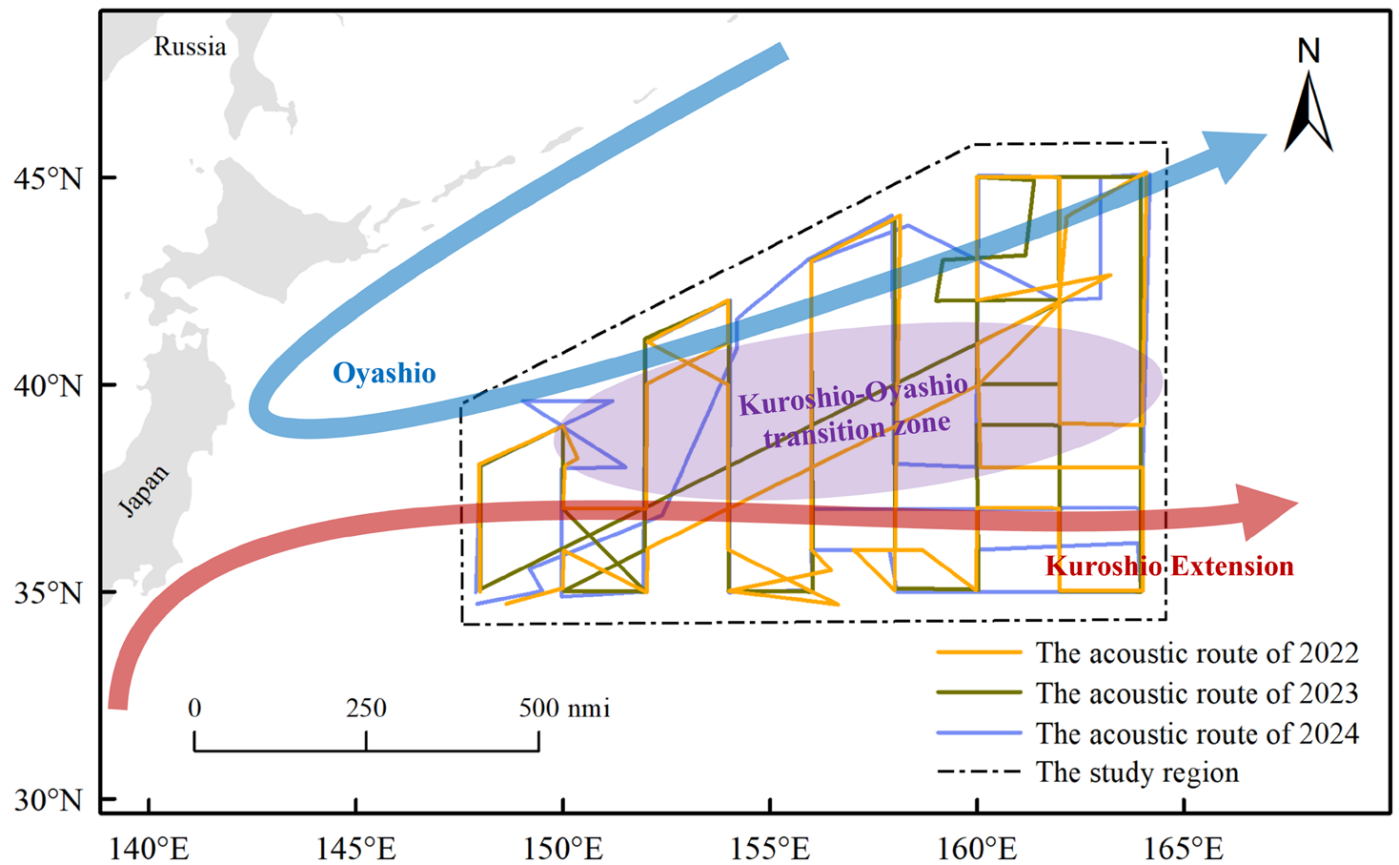
Survey tracks of the RV Songhang during 2022–2024, with missing data in some track segments.

The survey area was located in the Northwest Pacific Ocean, spanning 34°–45° N and 147°–165° E (Figure 1). Surveys were conducted from 15 June to 29 July 2022, from 16 June to 7 August 2023, and from 10 June to 2 August 2024. All surveys were carried out aboard the RV Songhang.

### 2.2 Data Collection

#### 2.2.1 Acoustic Data

Acoustic data were collected using Simrad EK80 (Kongsberg Gruppen ASA, Norway). The vessel speed during the transect survey was approximately 10 knots. Narrowband data were collected at 38, 70, 120, and 200 kHz (Table 1). Data at 38 kHz was used in this study to ensure consistency across years and across both surface SSL and DSL, because it provided the most complete and comparable depth coverage throughout the surveys. This frequency has been widely applied in SSL studies worldwide and has been shown to be effective (Fennell and Rose 2015; Proud et al. 2018; Kang et al. 2024). During the surveys, calibration was conducted using the standard sphere method (Demer et al. 2015) with a 38.1 mm tungsten carbide sphere (WC 38.1).

**TABLE 1:** Parameters of the EK80 scientific echosounder.

Parameter	38 kHz	70 kHz	120 kHz	200 kHz
Transducers	ES38‐7	ES70‐7C	ES120‐7C	ES200‐7C
Transmitting Power (W)	2000	750	250	150
Pulse Duration (ms)	1.024	1.024	1.024	1.024
Beam Angle (°)	7	7	7	7
Rampling Mode	Fast	Fast	Fast	Fast

#### 2.2.2 Environmental Data

RV Songhang collected CTD data at 78, 79, and 76 stations during the three annual cruises, respectively. However, due to limitations in data availability, this study did not use the CTD data and instead used satellite remote sensing data. The following ocean environmental variables were considered in this study, including sea surface temperature (SST, °C), sea surface salinity (SSS, psu), sea surface height (SSH, m), mixed layer depth (MLD, m), chlorophyll *a* concentration (Chl *a*, mg/m^3^), dissolved oxygen concentration (DO, mmol/m^3^), net primary production (NPP, mg/m^3^), upward seawater velocity (USWV, m/s), and eddy kinetic energy (EKE, m^2^/s^2^). These variables have been considered to influence the distribution of communities within the SSL in previous studies (Béhagle et al. 2016; Wang et al. 2019; Assunção et al. 2023; Nie et al. 2023; Wan and Chen 2024). Data for SST, SSS, SSH, MLD, USWV, Chl *a*, DO, and NPP were downloaded from the Copernicus Marine Environment Management Service platform [https://data.marine.copernicus.eu/products (accessed on 5 October 2025)]. The datasets were obtained from the Global Ocean Physics Analysis and Forecast product (spatial resolution 0.083°× 0.083°) and the Global Ocean Biogeochemistry Analysis and Forecast product (spatial resolution 0.25°× 0.25°), both provided at a daily temporal resolution. EKE was calculated as follows:

(1) $$\mathrm{EKE}=\frac{u^{2}+v^{2}}{2}$$

where u and v are the zonal and meridional geostrophic velocity anomalies, respectively. Both variables were downloaded from the Global Ocean Physics Analysis and Forecast product. The spatial resolution was 0.083°× 0.083°, and the temporal resolution was 1 day. The annual mean distributions of the environmental variables included in the optimal GAM for each year are shown in the Appendix.

### 2.3 Acoustic Data Noise Removal

Acoustic data were analyzed using Echoview 13.0 (Echoview Pty. Ltd., Australia). Acoustic data were analyzed over a depth range of 10–1000 m below the surface to exclude interference from near‐surface bubbles. Following the acoustic data processing framework proposed by Ryan et al. (2015), four noise removal algorithms (De Robertis and Higginbottom 2007; Ryan et al. 2015; Boswell et al. 2020) were applied sequentially to remove the effects of impulse noise, signal attenuation, transient noise, and background noise. Subsequently, visual inspection combined with manual editing was used to exclude noise that was not removed by algorithms. The elementary distance sampling unit (EDSU) was set to 24 h in the horizontal dimension and 1000 m in the vertical dimension. Using a daily time step, acoustic data of 10–1000 m were integrated. Daily mean volume backscattering strength (MVBS, dB) and the nautical area scattering coefficient (NASC, m^2^/nmi^2^) were calculated after noise removal.

### 2.4 SSL Descriptors

A complete MVBS dataset derived from echo‐integration was used to generate vertical profiles of acoustic backscatter. This dataset contained acoustic observations for each EDSU grid cell, defined as 24 h in the horizontal dimension and 1 m in the vertical dimension. For each year, the linear mean of all observations was calculated at each depth. This produced an annual vertical backscatter profile at a 1 m depth resolution. Spearman rank correlation coefficients were used to quantify the correlations among the annual vertical profile curves (Mouget et al. 2025).

SSL descriptors were used to characterize the aggregated spatial organization of pelagic communities. SSL were visually identified from daily echograms, and SSL regions were delineated using the horizontal box selection method. Based on these regions, multiple SSL descriptors were extracted to quantify SSL characteristics. These descriptors included those focused on SSLs and those derived from echo‐integration across the full water column (Perrot et al. 2018; Mouget et al. 2022, 2025). The latter capture the overall water column and incorporate biological information outside the SSL. They therefore complement descriptors derived specifically from SSLs. The SSL descriptors (see figure A1 in the Appendix) used in this study included (Weill et al. 1993; MacLennan et al. 2002; Woillez et al. 2007; Urmy et al. 2012; Mouget et al. 2022, 2025): (1) MVBS for the full echogram, describing overall scattering conditions in the water column; (2) MVBS of the shallowest SSL (${\text{MVBS}}_{1}$), capturing characteristics of the shallowest SSL; (3) MVBS of all SSLs (${\text{MVBS}}_{\mathrm{all}}$), providing an integrated description of SSLs across the water column; (4) echo‐integration of all SSLs (${\text{NASC}}_{\mathrm{all}}$), which was proportional to the acoustic density of communities within all SSLs in the water column; (5) mean depth of the shallowest SSL (${\text{MeanDepth}}_{1}$), representing the position of the shallowest SSL; (6) width of the shallowest SSL (${\text{Width}}_{1}$), which was related to the behavior of the shallowest SSL; (7) number of SSLs in the water column (N), reflecting the vertical structural complexity of pelagic communities. MVBS, ${\text{MVBS}}_{1}$, and ${\text{NASC}}_{\mathrm{all}}$ were derived from echo‐integration. ${\text{MeanDepth}}_{1}$, ${\text{Width}}_{1}$, and N were obtained using depth information and visual counts. ${\text{MVBS}}_{\mathrm{all}}$ was calculated as follows:

(2) $${\text{MVBS}}_{\mathrm{all}}=10\log_{10}\frac{\sum_{i=1}^{N}10^{\frac{{\text{MVBS}}_{i}}{10}}}{N}$$

where i denotes the SSL index, and ${\text{MVBS}}_{i}$ is the MVBS of the i th SSL.

For continuous descriptors, including MVBS, ${\text{MVBS}}_{1}$, ${\text{MVBS}}_{\mathrm{all}}$, ${\text{NASC}}_{\mathrm{all}}$, ${\text{MeanDepth}}_{1}$, and ${\text{Width}}_{1}$, kernel density estimation was used to construct probability density functions (Mouget et al. 2025). For the discrete descriptor N, only the frequency distribution was calculated. Analyses across descriptors enabled comparison of the relative importance of acoustic density inside and outside the SSL. They also allowed contrasts between the shallowest SSL and all SSLs, thereby revealing changes in water column structure.

### 2.5 Environmental Influence Analysis

#### 2.5.1 Matching Acoustic and Environmental Data

NASC is an acoustic index that effectively reflects the distribution of pelagic communities. It is commonly considered proportional to biomass within the survey area (MacLennan et al. 2002; Xue et al. 2025). Among the SSL descriptors, ${\text{NASC}}_{\mathrm{all}}$ provides the most integrative measure of the acoustic density of pelagic communities across the full water column. Therefore, ${\text{NASC}}_{\mathrm{all}}$ was selected as the response variable for environmental analyses.

${\text{NASC}}_{\mathrm{all}}$ and environmental data were resampled to a 0.25° × 0.25° grid. For each grid cell, resampled values of ${\text{NASC}}_{\mathrm{all}}$ and environmental variables were calculated as the mean of all original observations within that cell. Environmental data were then extracted and matched using the date and location of each ${\text{NASC}}_{\mathrm{all}}$ grid cell.

#### 2.5.2 GAM Analysis

GAM is a nonparametric multiple regression approach that can directly represent nonlinear relationships between a response variable and multiple explanatory variables (Xue et al. 2025). GAM was used to model year‐specific relationships between the distribution of pelagic communities and environmental factors. A full‐factor GAM was fitted with latitude (Lat), longitude (Lon), SST, SSS, SSH, MLD, USWV, Chl *a*, DO, NPP, and EKE as explanatory variables. The full model formulation was:

(3) $$\begin{array}{c} \ln{\text{NASC}}_{\mathrm{all}}=s\left(\mathrm{Lat}\right)+s\left(\mathrm{Lon}\right)+s\left(\mathrm{SST}\right)+s\left(\mathrm{SSS}\right)+s\left(\mathrm{SSH}\right)\\ \;+s\left(\mathrm{MLD}\right)+s\left(\text{USWV}\right)+s\left(\mathrm{Chl}\;a\right)+s\left(\mathrm{DO}\right)\\ \;+s\left(\mathrm{NPP}\right)+s\left(\mathrm{EKE}\right)+\varepsilon \end{array}$$

where s denotes a natural spline smoother, and ε represents the relative error.

Before fitting the GAM, variance inflation factors (VIF) were used to evaluate collinearity among explanatory variables (Zhu et al. 2024). Variables with VIF > 10 were considered to show multicollinearity with other predictors (Zhao et al. 2014; Robinson et al. 2021; Yang et al. 2022) and were excluded. All explanatory variables retained after VIF screening were then entered into the GAM using stepwise selection. Akaike information criterion (AIC) was used to balance goodness of fit and model complexity, yielding the optimal model (Planque et al. 2007). GAMs were fitted in RStudio 4.2.1 (RStudio PBC, America) using the mgcv package (Wood 2011).

## 3 Results

### 3.1 Vertical Backscatter Profiles

**FIGURE 2:**
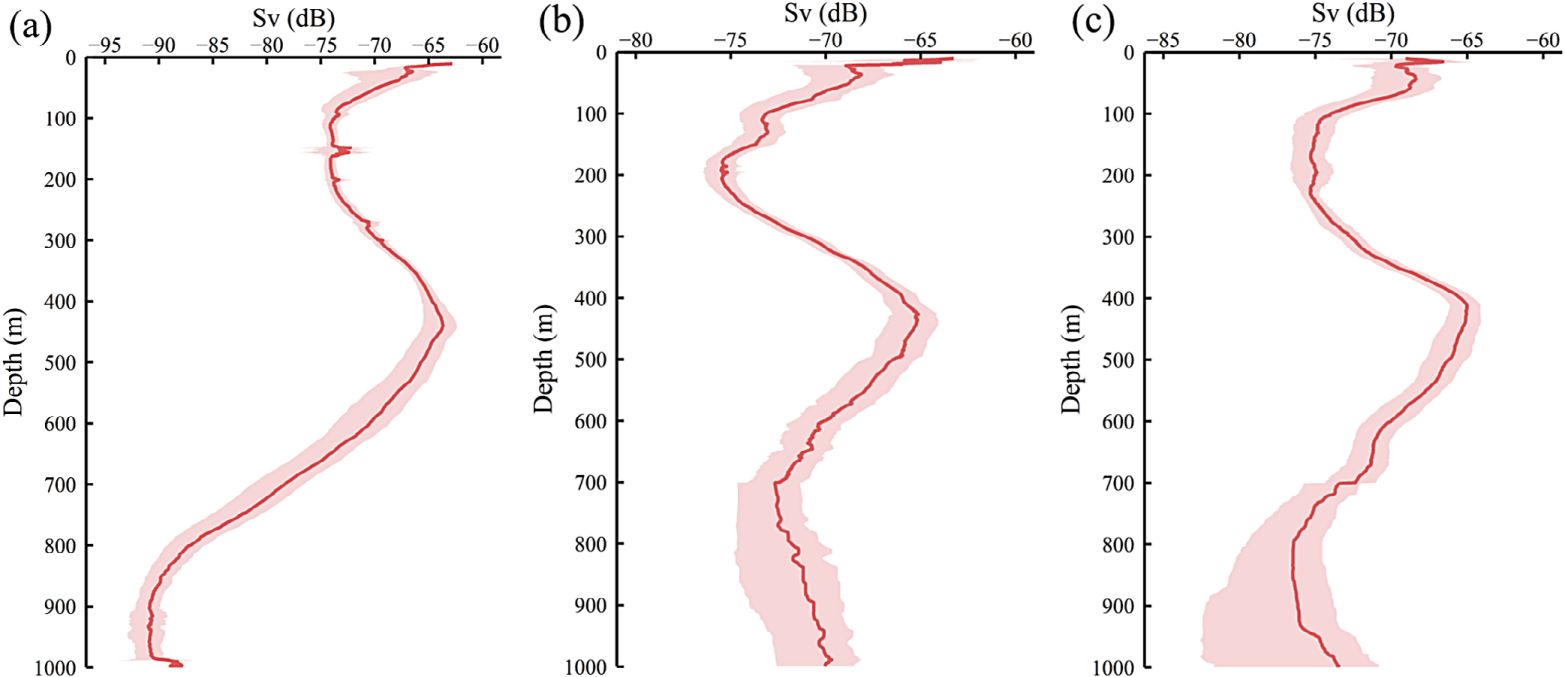
Vertical backscatter profiles in different years. (a) 2022; (b) 2023; (c) 2024.

Vertical backscatter profiles reflect the vertical structure of the SSL. Profiles of 3 years showed similar structures (Figure 2). Spearman rank correlation coefficients among the three profiles ranged from 0.59 to 0.87, indicating significant positive correlations (p < 0.05). Two persistent SSLs occurred throughout the year in the study area. A shallow SSL was located at 0–100 m. A deep scattering layer (DSL) was located at 300–700 m. Peak scattering strength in both SSLs was approximately −65 dB. In 2022, vertical variability in scattering strength was larger than in the other years. Scattering strength below 700 m was much weaker, whereas the DSL showed the greatest thickness among the 3 years. In 2023, scattering below 700 m was stronger and increased with depth. In 2024, the shallow SSL was relatively weak, and a subsurface SSL occurred near 50 m. Its scattering strength was slightly lower than that of the surface SSL. In addition, the DSL extended deeper in 2022 and 2024 than in 2023, reaching 800 m.

### 3.2 SSL Descriptors

**FIGURE 3:**
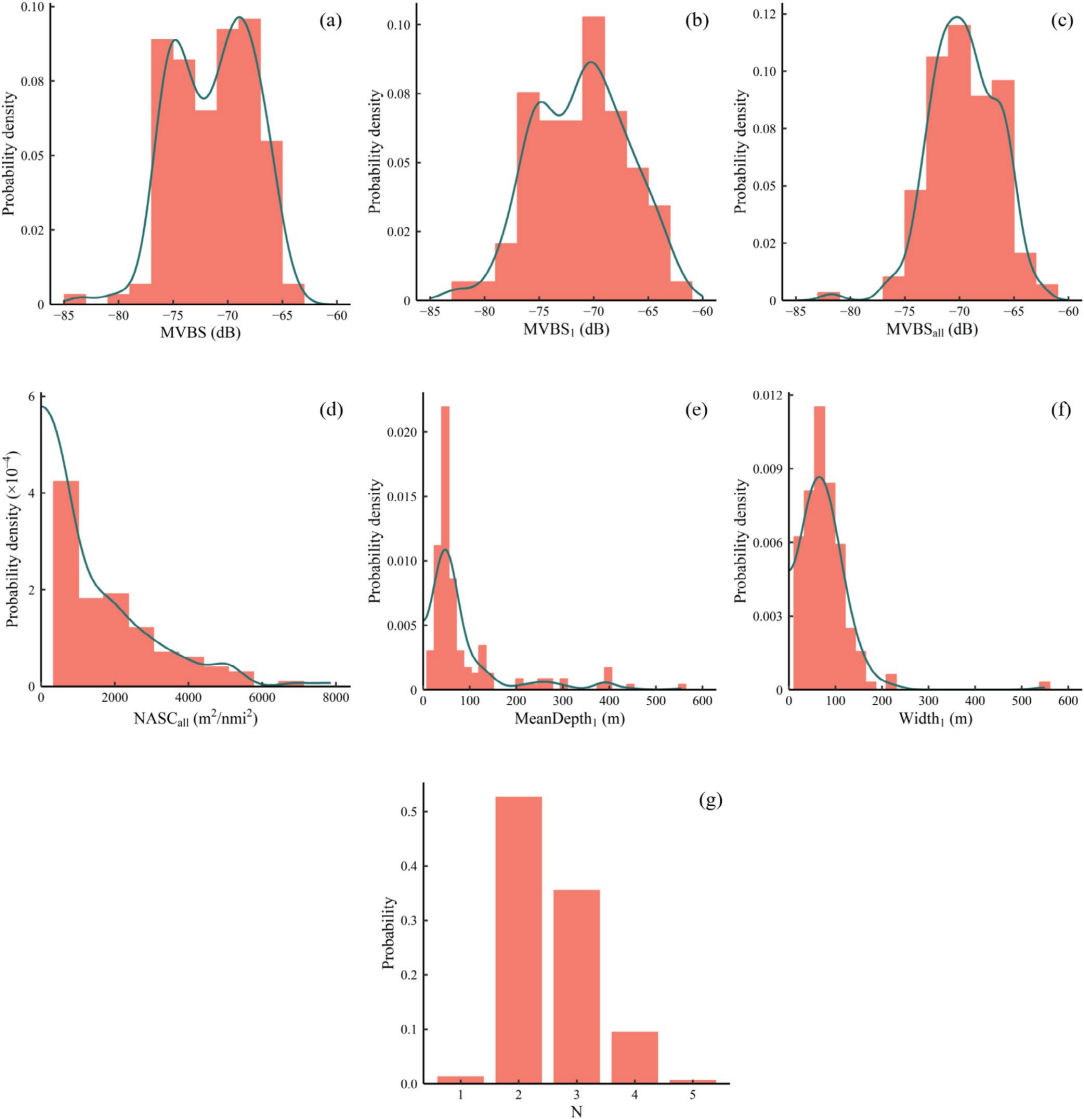
Kernel density curves or frequency distribution of SSL descriptors based on the data from 2022 to 2024. (a) MVBS; (b) ${\text{MVBS}}_{1}$; (c) ${\text{MVBS}}_{\mathrm{all}}$; (d) ${\text{NASC}}_{\mathrm{all}}$; (e) ${\text{MeanDepth}}_{1}$; (f) ${\text{Width}}_{1}$; (g) N.

Based on the kernel density curves and frequency distributions of SSL descriptors (Figure 3), the value ranges and concentration intervals differed markedly among descriptors. MVBS (Figure 3a) showed a bimodal distribution, with two high‐density modes centered at −76 ~ −68 dB. ${\text{MVBS}}_{1}$ (Figure 3b) showed a unimodal distribution with a broad spread across the full range, peaking at approximately −70 dB. ${\text{MVBS}}_{\mathrm{all}}$ (Figure 3c) showed an approximately symmetric unimodal distribution, with a peak also near −70 dB. ${\text{NASC}}_{\mathrm{all}}$ (Figure 3d) showed a strongly right‐skewed distribution with a heavy tail, with most values below 4000 m^2^/nmi^2^. Only a small number of transects exhibited extremely high NASC. ${\text{MeanDepth}}_{1}$ (Figure 3e) showed a right‐skewed distribution. The shallowest SSL occurred above 100 m in most cases, particularly near 50 m. In some profiles it occurred deeper than 150 m. ${\text{Width}}_{1}$ (Figure 3f) also showed a right‐skewed distribution, with a distinct peak at 50–100 m. Thicknesses exceeding 200 m were uncommon, indicating that the shallowest SSL was generally limited in vertical extent. N was most commonly two or three (Figure 3g), with probabilities of approximately 0.53 and 0.36, respectively. Only a small number of transects showed a single SSL or more than four SSLs.

### 3.3 Environmental Influence Analysis

#### 3.3.1 Spatiotemporal Distribution of the Original ${\text{NASC}}_{\mathrm{all}}$

**FIGURE 4:**
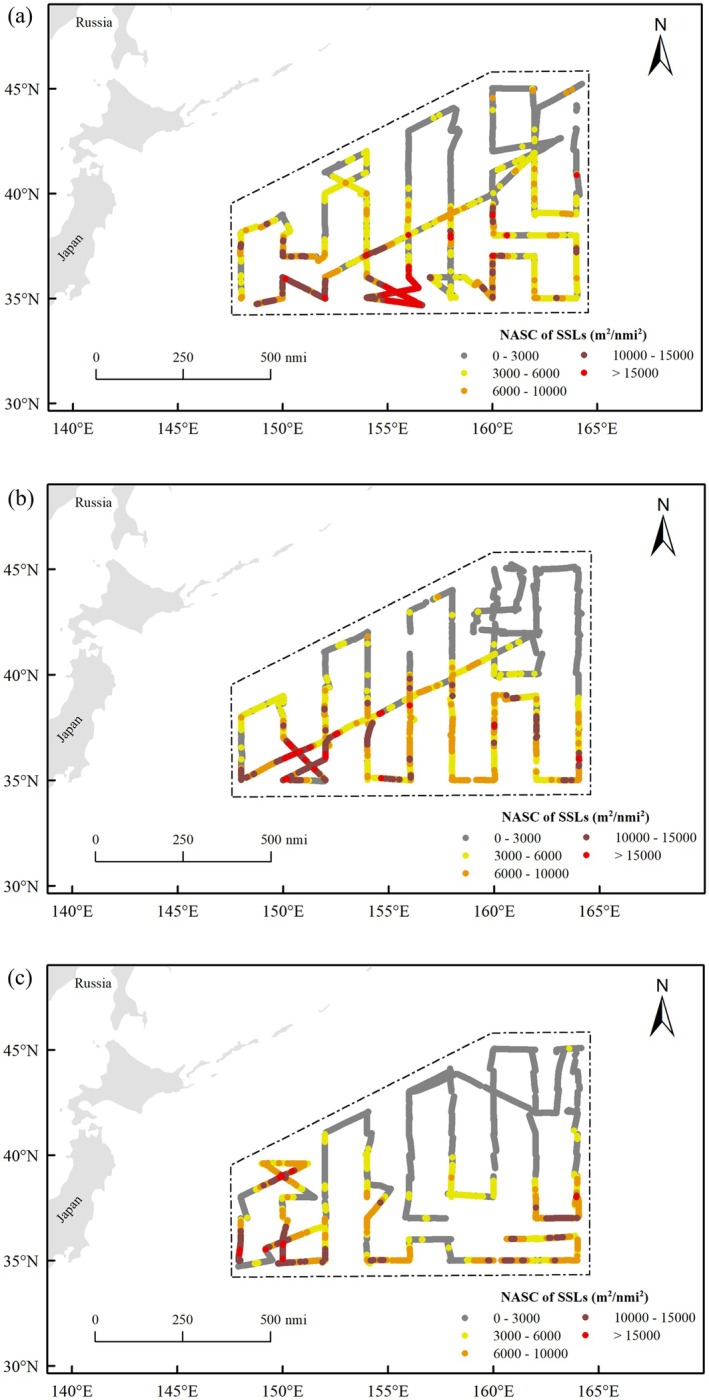
Distribution of ${\text{NASC}}_{\mathrm{all}}$ in different years. (a) 2022; (b) 2023; (c) 2024.

The mean ${\text{NASC}}_{\mathrm{all}}$ values were 4344.32 m^2^/nmi^2^ in 2022, 4186.75 m^2^/nmi^2^ in 2023, and 3546.97 m^2^/nmi^2^ in 2024. High NASC aggregation areas were broadly similar across the 3 years. They were mainly concentrated in the southwestern part of the study area at 34°–37° N, 148°–157° E, and in the southeastern at 34°–38° N, 160°–164° E (Figure 4). The maximum NASC in 2022 occurred at 38° N and 158° E, reaching 95846.8 m^2^/nmi^2^. In 2023, it occurred at 38.53° N and 156° E, reaching 36381.75 m^2^/nmi^2^. In 2024, it occurred at 35.52° N and 148.19° E, reaching 697774.71 m^2^/nmi^2^. Overall, acoustic density was higher in the southern region than in the northern.

#### 3.3.2 Collinearity Assessment of Environmental Variables

In 2022, 2023, and 2024, Chl *a* and DO consistently showed the highest VIF values and exceeded 10, whereas most other variables had VIF values below 10. After removing the two variables, VIF values for the remaining nine environmental variables were all below 10. Therefore, Lat, Lon, SST, SSS, SSH, MLD, USWV, NPP, and EKE were used to fit the GAM for each year.

#### 3.3.3 Optimal GAM Construction

The optimal GAM for 2022 was (Table 2):

(4) $$\begin{array}{c} \ln{\text{NASC}}_{\mathrm{all}}=s\left(\mathrm{SSH}\right)+s\left(\mathrm{SST}\right)+s\left(\mathrm{Lon}\right)+s\left(\mathrm{Lat}\right)\\ \;+s\left(\mathrm{SSS}\right)+s\left(\mathrm{NPP}\right)+s\left(\mathrm{MLD}\right) \end{array}$$

**FIGURE 5:**
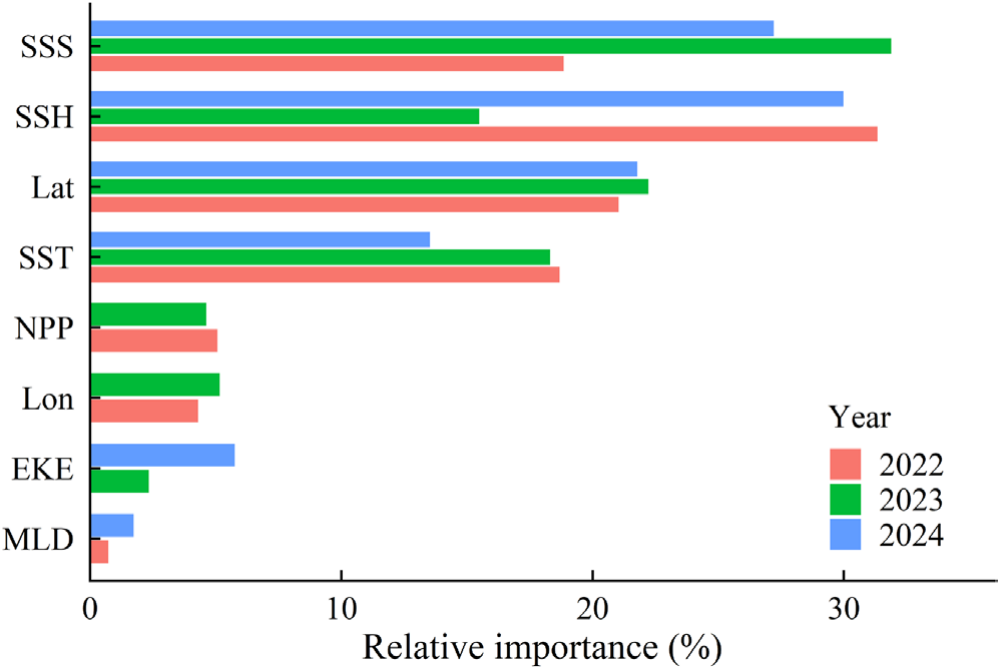
Relative importance of optimal GAMs of different years.

The optimal GAM had an AIC of 417.02, an explained deviance of 86.1%, and an $R^{2}$ of 0.85. SSH, SST, Lon, and Lat were highly significantly associated with ${\text{NASC}}_{\mathrm{all}}$ (p < 0.001). Relative importance of explanatory variables was quantified using the relative weight approach proposed by J. W. Johnson (2000). The relative importance values of SSH, Lat, SSS, SST, NPP, Lon, and MLD were 31.34%, 21.03%, 18.85%, 18.68%, 5.07%, 4.3%, and 0.73%, respectively (Figure 5).

**TABLE 2:** The parameters of optimal GAMs.

Year	Models	Variables	*F*	p	Deviance explanation	AIC	$R^{2}$
2022	$\ln{\text{NASC}}_{\mathrm{all}}=s\left(\mathrm{SSH}\right)+s\left(\mathrm{SST}\right)+s\left(\mathrm{Lon}\right)+s\left(\mathrm{Lat}\right)+s\left(\mathrm{SSS}\right)+s\left(\mathrm{NPP}\right)+s\left(\text{USWV}\right)$	SSH	39.694	< 0.001	85.9%	421.24	0.848
		SST	6.283	< 0.001			
		Lon	8.489	< 0.001			
		Lat	4.361	< 0.001			
		SSS	2.267	< 0.05			
		NPP	1.712	0.079			
		USWV	0.658	0.418			
2023	$\ln{\text{NASC}}_{\mathrm{all}}=s\left(\mathrm{SSS}\right)+s\left(\mathrm{SST}\right)+s\left(\mathrm{SSH}\right)+s\left(\mathrm{Lat}\right)+s\left(\mathrm{NPP}\right)+s\left(\mathrm{EKE}\right)+s\left(\text{USWV}\right)$	SSS	4.983	< 0.001	81.4%	802.97	0.804
		SST	11.041	< 0.001			
		SSH	11.146	< 0.001			
		Lat	8.728	< 0.001			
		NPP	4.793	< 0.001			
		EKE	4.259	< 0.001			
		USWV	0.023	0.88			
2024	$\ln{\text{NASC}}_{\mathrm{all}}=s\left(\mathrm{SST}\right)+s\left(\mathrm{SSH}\right)+s\left(\mathrm{Lat}\right)+s\left(\mathrm{SSS}\right)+s\left(\mathrm{EKE}\right)+s\left(\text{USWV}\right)$	SST	20.054	< 0.001	76.7%	1030.81	0.748
		SSH	11.662	< 0.001			
		Lat	4.852	< 0.001			
		SSS	3.909	< 0.001			
		EKE	3.043	< 0.005			
		USWV	0.315	0.591			

The optimal GAM for 2023 was (Table 2):

(5) $$\begin{array}{c} \ln{\text{NASC}}_{\mathrm{all}}=s\left(\mathrm{SSS}\right)+s\left(\mathrm{SST}\right)+s\left(\mathrm{SSH}\right)+s\left(\mathrm{Lat}\right)\\ \;+s\left(\mathrm{NPP}\right)+s\left(\mathrm{EKE}\right)+s\left(\mathrm{Lon}\right) \end{array}$$

This GAM had an AIC of 787.06, an explained deviance of 82.4%, and an $R^{2}$ of 0.81. SSS, SST, SSH, Lat, NPP, and EKE were highly significantly associated with ${\text{NASC}}_{\mathrm{all}}$ (p< 0.001), and Lon was significantly associated with ${\text{NASC}}_{\mathrm{all}}$ (p< 0.005). The relative importance values of SSS, Lat, SST, SSH, Lon, NPP, and EKE were 31.88%, 22.22%, 18.3%, 15.49%, 5.15%, 4.62%, and 2.34%, respectively (Figure 5).

The optimal GAM for 2024 was (Table 2):

(6) $$\ln{\text{NASC}}_{\mathrm{all}}=s\left(\mathrm{SST}\right)+s\left(\mathrm{SSH}\right)+s\left(\mathrm{Lat}\right)+s\left(\mathrm{SSS}\right)+s\left(\mathrm{EKE}\right)+s\left(\mathrm{MLD}\right)$$

It had an AIC of 1018.06, an explained deviance of 77.4%, and an $R^{2}$ of 0.76. SSS, SST, SSH, Lat, and EKE were highly significantly associated with ${\text{NASC}}_{\mathrm{all}}$ (p< 0.001), and MLD was significantly associated with ${\text{NASC}}_{\mathrm{all}}$ (p< 0.005). The relative importance values of SSH, SSS, Lat, SST, EKE, and MLD were 29.98%, 27.21%, 21.78%, 13.53%, 5.76%, and 1.73%, respectively (Figure 5).

#### 3.3.4 Relationships Between ${\text{NASC}}_{\mathrm{all}}$ and Environmental Variables

**FIGURE 6:**
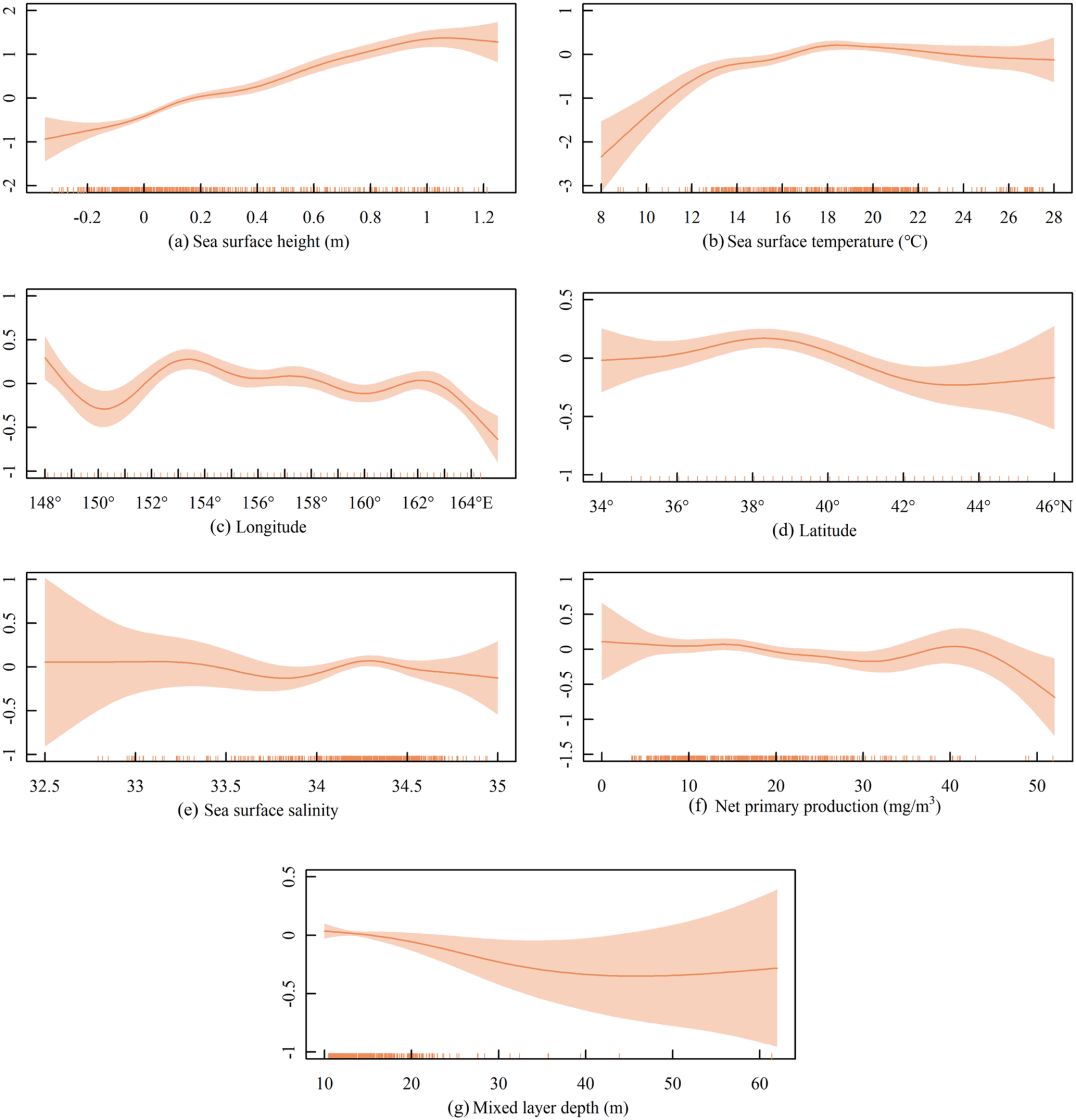
Relationships between environmental variables and ${\text{NASC}}_{\mathrm{all}}$ in the optimal GAM for 2022. (a) SSH; (b) SST; (c) Lon; (d) Lat; (e) SSS; (f) NPP; (g) MLD.

In 2022, ${\text{NASC}}_{\mathrm{all}}$ showed a monotonic increasing relationship with SSH. It increased continuously with SSH from approximately −0.2 m onward, and then approached a stable level when SSH reached 1 m (Figure 6a). The relationship between ${\text{NASC}}_{\mathrm{all}}$ and SST showed a rapid increase followed by a plateau. It increased sharply at 8°C–12°C and remained stable at 18°C–22°C, with little change at the warm end (Figure 6b). Relationships with Lon and Lat were nonlinear and showed multiple peaks (Figure 6c,d). ${\text{NASC}}_{\mathrm{all}}$ varied little with SSS. Only minor fluctuations occurred at 33.5–34.5 (Figure 6e). ${\text{NASC}}_{\mathrm{all}}$ tended to decrease as NPP increased. But it increased monotonically at 35–40 mg/m^3^ and then declined (Figure 6f). ${\text{NASC}}_{\mathrm{all}}$ showed a negative relationship with MLD at 10–35 m. Beyond this range, a stable increase occurred as limited samples (Figure 6g).

**FIGURE 7:**
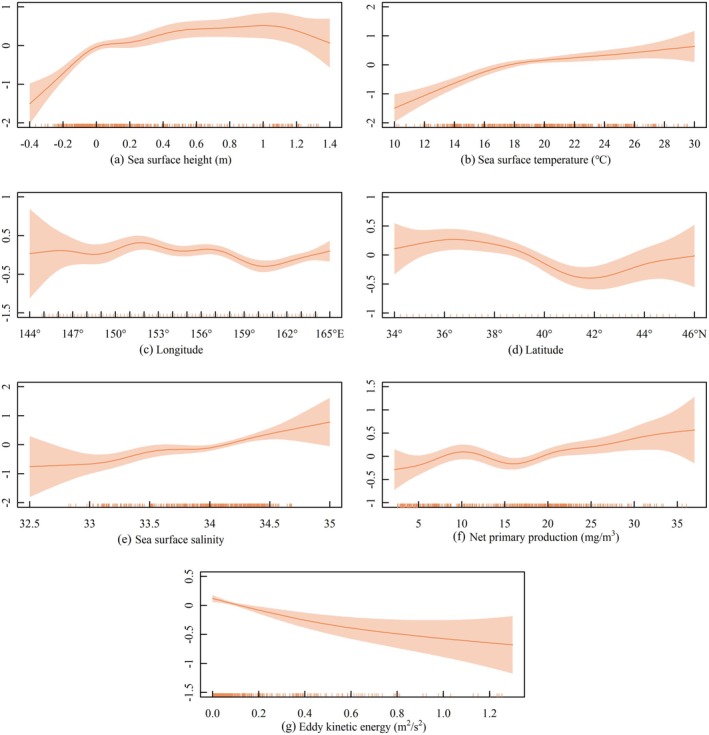
Relationships between environmental variables and ${\text{NASC}}_{\mathrm{all}}$ in the optimal GAM for 2023. (a) SSH; (b) SST; (c) Lon; (d) Lat; (e) SSS; (f) NPP; (g) EKE.

In 2023, the relationship between SSH and ${\text{NASC}}_{\mathrm{all}}$ showed a rapid increase followed by a slower increase and then a decline (Figure 7a). ${\text{NASC}}_{\mathrm{all}}$ increased most rapidly when SSH ranged from −0.4 m to 0 m. The effect of SST was also increasing (Figure 7b). ${\text{NASC}}_{\mathrm{all}}$ increased markedly at 10°C–18°C and leveled off after 20°C. The relationship with Lon was similar to that in 2022 and showed a multi‐peaked nonlinear pattern (Figure 7c). However, fluctuations in ${\text{NASC}}_{\mathrm{all}}$ along Lon were small in 2023, and no clear monotonic trend was evident. The relationship with Lat showed an approximately sinusoidal pattern (Figure 7d). ${\text{NASC}}_{\mathrm{all}}$ showed an increasing relationship with SSS, with only slight fluctuations between 33 and 34 (Figure 7e). ${\text{NASC}}_{\mathrm{all}}$ increased with fluctuations as NPP increased (Figure 7f). It had a slight decline occurred around 10–15 mg/m^3^, and then increased at a lower slope. EKE showed a stable negative relationship with ${\text{NASC}}_{\mathrm{all}}$. ${\text{NASC}}_{\mathrm{all}}$ decreased approximately linearly as EKE increased (Figure 7g).

**FIGURE 8:**
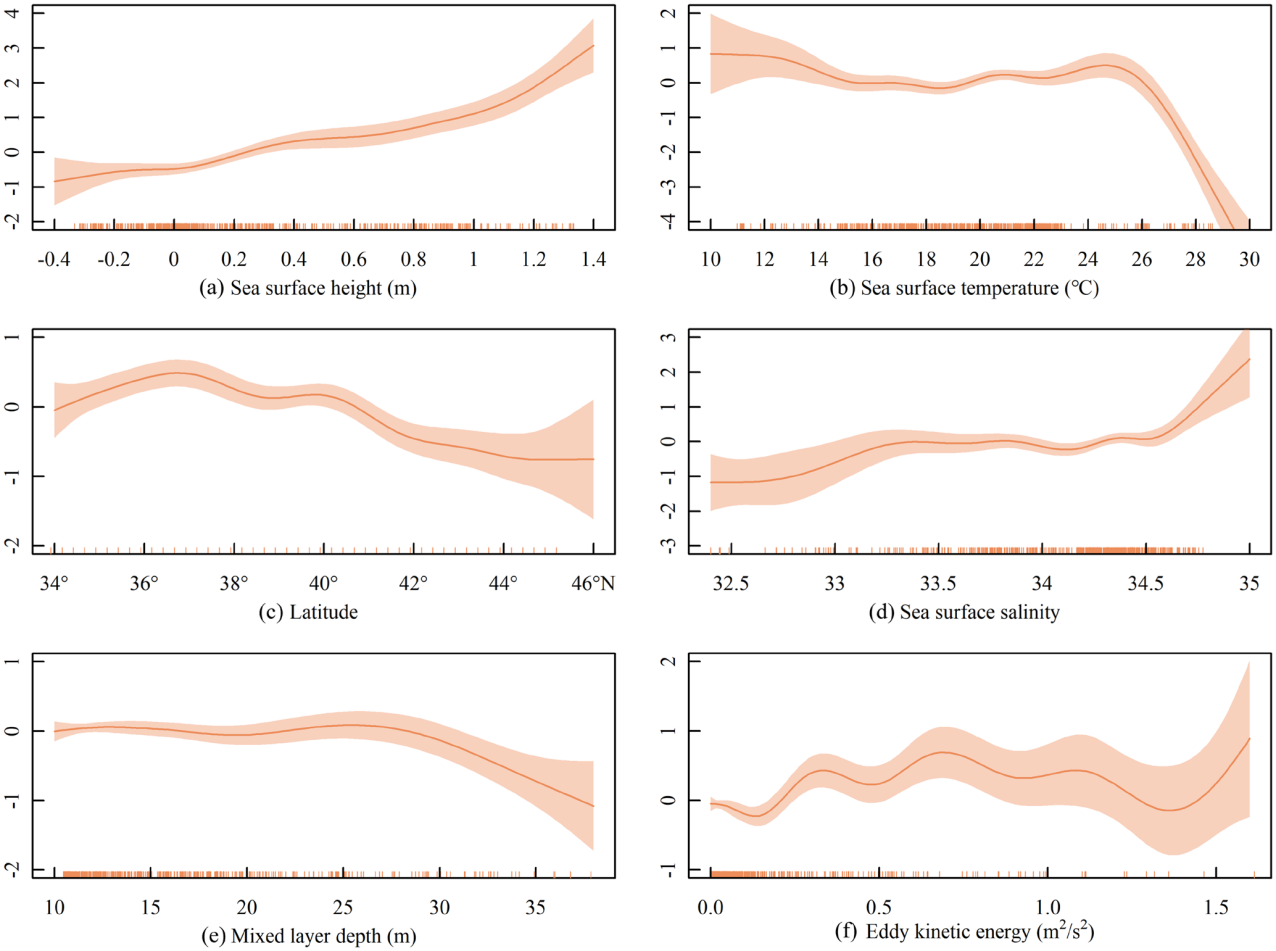
Relationships between environmental variables and ${\text{NASC}}_{\mathrm{all}}$ in the optimal GAM for 2024. (a) SSH; (b) SST; (c) Lat; (d) SSS; (e) MLD; (f) EKE.

In 2024, the effect of SSH on ${\text{NASC}}_{\mathrm{all}}$ showed an increasing nonlinear relationship (Figure 8a). The increase was relatively gradual when SSH ranged from −0.4 to 1.1 m and became steeper when it exceeded 1.2 m. ${\text{NASC}}_{\mathrm{all}}$ changed little as SST increased at 10°C–24°C, with only minor fluctuations. When SST exceeded approximately 25°C, ${\text{NASC}}_{\mathrm{all}}$ declined sharply (Figure 8b). The relationship between ${\text{NASC}}_{\mathrm{all}}$ and Lat showed an increase at 34°–37° N, followed by a gradual decline (Figure 8c). The effect of SSS on ${\text{NASC}}_{\mathrm{all}}$ showed an opposite pattern to that of SST (Figure 8d). ${\text{NASC}}_{\mathrm{all}}$ increased gradually as SSS rose from 32.5 to 33.3. ${\text{NASC}}_{\mathrm{all}}$ then fluctuated slightly between 33.5 and 34.5. When SSS exceeded 34.5, ${\text{NASC}}_{\mathrm{all}}$ increased sharply. The effect of MLD on ${\text{NASC}}_{\mathrm{all}}$ was similar to that of SST (Figure 8e). ${\text{NASC}}_{\mathrm{all}}$ changed little as MLD increased from 10 m to 25 m and then decreased as MLD deepened. Although ${\text{NASC}}_{\mathrm{all}}$ showed a clear multi‐peaked nonlinear relationship with EKE, overall variation remained within a limited range, and no consistent increasing or decreasing trend was evident (Figure 8f).

Overall (Table 3), SSH showed the most consistently positive effect across the 3 years, although the exact shape of the response curve differed among years. SST generally showed a threshold‐like relationship, with increases over an intermediate temperature range followed by stabilization or decline at the warm end. In contrast, Lat and Lon mainly exhibited multi‐peaked nonlinear relationships, suggesting that geographic position primarily reflected spatially structured heterogeneity rather than simple monotonic gradients. The effects of SSS, NPP, MLD, and EKE were less consistent among years, indicating stronger interannual dependence in their influence on ${\text{NASC}}_{\mathrm{all}}$.

**TABLE 3:** Relationships between environmental variables and ${\text{NASC}}_{\mathrm{all}}$ in the optimal GAMs.

Variable	Year		
	2022	2023	2024
SSH	Increasing, then plateau	Rapid increase, then slower increase and decline	Nonlinear increase
SST	Rapid increase, then plateau	Increase, then plateau	Stable over intermediate range, then sharp decline at high end
SSS	Week fluctuations	Increasing	Increasing at high salinity end
Lat	Multi‐peaked nonlinear	Approximately sinusoidal	Increase, then decline
Lon	Multi‐peaked nonlinear	Weak multi‐peaked nonlinear	—
NPP	Mostly negative with local increase	Fluctuating increase	—
MLD	Negative at shallow range, then slight increase under limited samples	—	Stable, then decreasing
EKE	—	Negative	Multi‐peaked, weak overall variation

## 4 Discussion

### 4.1 Spatiotemporal Distribution Patterns of Pelagic Communities

The SSL in the Northwest Pacific Ocean comprises dense assemblages of zooplankton and small to medium‐sized fishes (Fu et al. 2022) and supports high biodiversity. Zooplankton communities are dominated by Copepoda and planktonic larvae (Fu et al. 2022). The fish community is dominated by Myctophidae, *Scomber japonicus* , *Sardinops melanostictus* , and *Engraulis japonicus* (Zhu et al. 2024), together with amounts of small Cephalopoda. Spatiotemporal patterns in ${\text{NASC}}_{\mathrm{all}}$ effectively reflect the distribution of these pelagic communities. Although multifrequency or broadband features combined with biological sampling data can be further used for scattering‐group discrimination or species identification (Misund et al. 2025; Fréon and Misund 1999), such approaches focus on community composition and classification attribution, which are beyond the objective of this study. Overall, ${\text{NASC}}_{\mathrm{all}}$ showed recurring regional contrasts among years. High‐density aggregation areas were broadly similar over the three years (Figure 4). These areas largely follow the path of the Kuroshio Extension. Relatively warm waters and elevated primary production provide favorable conditions for the aggregation of aquatic organisms. Widespread fronts and eddies provide important recruitment habitats for taxa (Durán‐Campos et al. 2019), thereby promoting their aggregation. In addition, a smaller number of high‐density aggregation areas occurred in the central part of the study region, within the Kuroshio‐Oyashio transition zone. Numerous anticyclonic eddies occur near this zone, generating downwelling in their cores and upwelling along their peripheries (Liu et al. 2022). This configuration promotes nutrient exchange and enhances prey availability (Sugisaki and Kurita 2004). In the northeastern part of the study area, conditions are colder and fresher on the Oyashio side, which is less favorable for zooplankton (Aoki and Inagaki 1992). Pelagic communities were therefore less abundant in this region. This spatial pattern is consistent with the GAM results, in which Lat, SSS, and SSH were identified as important factors of pelagic community distribution, suggesting an important role of regional hydrographic structure in shaping SSL distribution.

The presence of a persistent shallow SSL and a DSL (Figure 2) indicates that dense pelagic communities are maintained over long periods in both the upper and deep layers. This two‐layer structure is consistent with the widespread co‐occurrence of shallow SSL and DSL in the global ocean. It reflects vertical niche differentiation of communities in relation to illumination, predation pressure, and food‐resource gradients. Illumination is one of the primary drivers of SSL migration and serves as one of the direct factors behind the migration of the SSL. It provides a temporal signal that triggers DVM. With sunrise and sunset as well as changes in solar altitude, illumination in the water column exhibits a distinct vertical gradient. Plankton are sensitive to light and exhibit phototaxis. They adjust their vertical distribution by perceiving both the absolute and relative rates of change in illumination, thereby maintaining themselves within an optimal illumination environment (Røstad et al. 2016). DVM is also a defensive mechanism and adaptive behavior developed in response to predation pressure (Benoit‐bird et al. 2017). Zooplankton avoid visual predators by shifting their depth of residence between day and night, effectively balancing the trade‐off between foraging benefits and predation risk. In addition, a thermocline commonly occurs at depths of 20–40 m in the Northwest Pacific Ocean (Xue et al. 2021). The thermocline acts as a barrier, limiting vertical exchange and restricting the downward penetration of warmer surface waters. Chl *a* and DO can form subsurface maxima near the thermocline, and pelagic communities often concentrate in this layer. This mechanism may explain why peaks in the vertical profiles occurred near this depth in each year. Overall, the shallow SSL and the DSL maintained stable basic structures over the 3 years (Figure 2). However, pronounced interannual differences were observed in DSL thickness, the depth of the lower boundary, and scattering strength below 700 m (Figure 2). These differences may reflect variability in midwater environmental conditions, community composition, and pelagic community responses to interannual oceanographic change. In 2024, the shallow SSL was slightly weaker overall (Figure 2c), but a subsurface SSL near 50 m was stronger than in the other 2 years (Figure 2a,b). This pattern may be related to the hypothesis that different species assemblages can exhibit similar structural organization under comparable environmental conditions (Schickele et al. 2020).

### 4.2 SSL Descriptors

SSL descriptors perform well in monitoring SSLs and zooplankton communities (Mouget et al. 2022). Descriptors derived from echo‐integration across the full water column complemented those focused specifically on SSLs. The descriptor MVBS derived from echo‐integration across the full water column enables classification and comparison based on the acoustic responses of different biotic communities (Mouget et al. 2025). Using MVBS provides a more comprehensive representation but typically involves greater computational effort. In contrast, descriptors focused on SSLs are less computationally demanding and are suitable for routine application. They can be used to compare and monitor non‐specific spatial structure of pelagic communities in marine ecosystems (Mouget et al. 2025).

In the Northwest Pacific Ocean, scattering strength values associated with SSLs typically range from −95 dB to −50 dB (Batzler and Vent 1967). Values above −70 dB are commonly associated with larger organisms such as pelagic fishes, whereas lower values generally indicate zooplankton (Diogoul et al. 2021). This interpretation is consistent with the concentration of MVBS, ${\text{MVBS}}_{1}$, and ${\text{MVBS}}_{\mathrm{all}}$ at approximately −80 dB to −65 dB in this study (Figure 3a–c). The bimodal pattern of MVBS suggests the presence of two dominant assemblage states in the water column, with shifts in their relative aggregation over time. When the water column is dominated by aggregations of weak scatterers, such as small zooplankton, MVBS tends to be lower. When more nekton is present, particularly swimbladdered fish or dense aggregations of mesopelagic fishes, MVBS increases markedly, giving rise to the second mode. ${\text{MVBS}}_{1}$ from the shallowest SSL was distributed broadly across −85 dB to −60 dB, with weaker concentration than MVBS and ${\text{MVBS}}_{\mathrm{all}}$. This pattern suggests that surface‐layer communities were more complex and heterogeneous. This pattern may be driven by increased taxonomic diversity or by a wider range of movement directions associated with feeding behavior (Proud et al. 2015). Neither exceptionally strong nor exceptionally weak layers consistently dominated the SSLs. Overall backscatter in the water column was mainly contributed by layers of moderate strength. This pattern suggests that SSLs in this region form a relatively stable acoustic background structure. Therefore, at broad spatial and temporal scales, MVBS, ${\text{MVBS}}_{1}$, and ${\text{MVBS}}_{\mathrm{all}}$ can be regarded as robust descriptors of the mean state of pelagic communities in the Northwest Pacific Ocean. The strongly right‐skewed, heavy‐tailed distribution of ${\text{NASC}}_{\mathrm{all}}$ (Figure 3d) indicates that overall biomass is strongly controlled by patchy aggregations of pelagic communities. This pattern is consistent with evidence that mesopelagic fishes and large zooplankton often form high‐density aggregations near fronts, along eddy margins, and in locally productive zones (Fennell and Rose 2015; Peña 2024).

The left‐skewed distribution of ${\text{MeanDepth}}_{1}$ and the predominance of 2–3 SSLs in the water column (Figure 3e,g) are consistent with SSL characteristics reported for most ocean regions worldwide. The higher‐density ranges of ${\text{MeanDepth}}_{1}$ at 0–100 m and 300–500 m also matched the surface and midwater bands in the vertical backscatter profiles (Figure 2). In most transects, the surface SSL shallower than 100 m served as the shallowest layer. The DSL became the shallowest layer only where the surface SSL was locally absent. This pattern suggests a strong dependence of surface‐layer communities on favorable environmental conditions. When surface conditions become unfavorable, pelagic communities tend to aggregate at greater depths. SSL thickness is highly sensitive to water mass structure and to environmental factors such as illumination and DO. When the oxygen minimum layer intensifies or illumination increases, the vertical extent of the SSL is often compressed. Under more permissive water‐mass conditions, the SSL can expand upward or downward (Aksnes et al. 2017; Diogoul et al. 2020). Therefore, variation in ${\text{Width}}_{1}$ in this study (Figure 3f) likely reflects expansion or compression of the habitat space available to pelagic communities under different water masses and environmental constraints.

The seven SSL descriptors used in this study helped characterize pelagic community distributions in waters influenced by the Kuroshio and the Oyashio. However, adjustments may be required for studies in other specific settings. This approach can be used to monitor the functional status of pelagic habitats (Cade and Benoit‐Bird 2014). It can also support understanding of pelagic community dynamics from a bioecological perspective (Proud et al. 2015). In situations where data availability is limited and biological validation data are not directly available for the analyzed acoustic observations, the standardized extraction of long time‐series of SSL descriptors may play a key role in analyzing temporal changes in pelagic communities. Comparative analyses of SSL descriptors across regions, habitats, and ecosystems can also help distinguish differences within and among pelagic ecosystems (Mouget et al. 2022) in the future.

### 4.3 Effects of Environmental Variability on the Distribution of Pelagic Communities

Over the years, Lat, SST, SSH, and SSS were key factors shaping the distribution of pelagic communities. Given the characteristics of the study region, the effect of Lat on pelagic communities was mainly linked to the Kuroshio Extension and the Oyashio. Water mass properties, frontal positions, and eddy activity change systematically with latitude. This interpretation is consistent with the oscillatory response of ${\text{NASC}}_{\mathrm{all}}$ along with the Lat. Temperature and salinity are key ecological factors in the ocean. They influence diversity, community composition, and spatial distribution. They also regulate physiological and ecological processes in zooplankton, thereby playing an important role in shaping community structure and food‐web organization (Ma et al. 2022). Temperature influences growth and reproduction in aquatic organisms. Temperatures that are too high or too low can suppress zooplankton development and reproductive processes. SSL backscatter strength has been reported to show a strong positive association with temperature at the depth of the layer (Proud et al. 2017). In this study, ${\text{NASC}}_{\mathrm{all}}$ showed a positive association with SST in 2022 and 2023, but no clear positive relationship was evident in 2024. This may be partly because SST was the only temperature metric used in this study, rather than temperature at depth. On the other hand, the DSL was thicker and extended deeper in 2024. Under strongly stratified conditions, its vertical position may have been more influenced by distinct midwater masses. SST was therefore less representative of the integrated acoustic density than in the other two years. In waters influenced by the Kuroshio and the Oyashio, variability in SSS is more informative as an indicator of the distribution of distinct water masses (Yasuda 2003). Different SSS ranges often correspond to the spatial distribution of the Kuroshio water, the Oyashio water, and mixed water masses. Notably, SSS was the most important predictor in 2023 based on relative importance. This is because high ${\text{NASC}}_{\mathrm{all}}$ areas overlapped closely with zones of moderately elevated SSS in 2023, which made SSS appear more important in the model. This result suggests that water mass structure and the confluence pattern in 2023 likely played a central role in shaping the distribution of pelagic communities. In contrast, SSH was the most important predictor in 2022 and 2024. SSH is closely linked to ocean dynamic processes such as eddies, fronts, and convergence (Alves et al. 2001; Kuroda and Yokouchi 2017). Eddies are widespread in the study region, and SSH differs between the interior and exterior of eddies. Variability in SSH also indicates large‐scale circulation and associated nutrient transport processes (Ayers and Lozier 2010; Xue et al. 2025). These dynamic processes can trigger or intensify aggregation of zooplankton and mesopelagic fishes over relatively short time scales, which helps explain why SSH accounted for more variation in SSL strength than SSS in 2022 and 2024. Lon, NPP, MLD, and EKE were less important but still showed measurable effects on SSL distribution. Overall, the distribution patterns of pelagic communities arise from the coupled influences of multiple environmental drivers (Figures A2–A4).

Because the study region encompasses the Kuroshio Extension, the Oyashio, and the Kuroshio‐Oyashio transition zone, ocean dynamic processes inevitably represent key drivers of spatiotemporal variability in pelagic communities. Most small to medium‐sized zooplankton that make up the SSL have limited swimming capacity and are readily influenced by currents and eddies. Mesoscale processes in the study region, including mesoscale eddies and fronts, can enhance energy transfer through the food web and promote aggregation of pelagic communities (Watson et al. 2018; Wan and Chen 2024). Understanding links between the distribution of small to medium‐sized zooplankton and ocean dynamic processes will help clarify the mechanisms driving pelagic community distributions at finer scales. This study examined SSS, SSH, and EKE as environmental variables, partially addressing the role of ocean dynamics. Further work is needed to investigate the underlying mechanisms in greater depth.

Illumination, an important factor influencing SSL behavior, was not included in the models. This is because its effects on the SSL are primarily expressed through DVM and vertical positioning. This study focused primarily on the horizontal distribution of the SSL. Spatiotemporal variability in illumination was partly represented indirectly by variables such as Lat and SST. In addition, the response variable used in the GAMs was defined as the total echo‐integration of all SSLs within 10–1000 m. Effects of illumination mediated through DVM and changes in SSL vertical depth were therefore integrated into this depth‐integrated metric. In addition, the GAMs showed high explained deviance in all years, indicating that the selected environmental variables captured key features of pelagic community distributions in the study region. Nevertheless, multi‐frequency analyses combined with biological sampling directly matched to acoustic observations (Misund et al. 2025; Fréon and Misund 1999) would improve sound scattering discrimination in future studies. Future work should apply finer vertical stratification to integrate horizontal distribution patterns with vertical structure. In addition, examining the environmental responses of other SSL descriptors (Diogoul et al. 2020) would be an important direction for future research. This three‐dimensional perspective will improve understanding of environmental responses in pelagic communities.

## 5 Conclusions

This study used SSL descriptors to analyze the distribution of pelagic communities in the Northwest Pacific Ocean and their environmental drivers. The results suggest that two dominant assemblage states may occur in the water column, with shifts over time in their relative aggregation. Pelagic community aggregation density was higher in the south than in the north. The distribution of communities was shaped by the combined effects of multiple environmental factors. Lat, SST, SSH, and SSS were identified as key drivers. These findings indicate that SSL descriptors are effective for monitoring and analyzing large‐scale aggregations of pelagic communities. This study provides a basis for investigating spatiotemporal variability in the pelagic ecosystem of the Northwest Pacific Ocean and offers a reference for further applications of SSL descriptors.

## Author Contributions

**Minghua Xue:** formal analysis (equal), investigation (equal), methodology (equal), software (equal), writing – original draft (equal). **Jianfeng Tong:** conceptualization (equal), funding acquisition (equal), supervision (equal), writing – review and editing (equal).

## Funding

This work was supported by National Key Research and Development Program of China (2023YFD2401302).

## Conflicts of Interest

The authors declare no conflicts of interest.

## Supporting information


**Figure SA1:** SSL descriptors used in this study.
**Figure SA2:** Distribution of environmental variables in the optimal GAM for 2022. (a) SSH; (b) SST; (c) SSS; (d) NPP; (e) MLD.
**Figure SA3:** Distribution of environmental variables in the optimal GAM for 2023. (a) SSH; (b) SST; (c) SSS; (d) NPP; (e) EKE.
**Figure SA4:** Distribution of environmental variables in the optimal GAM for 2024. (a) SSH; (b) SST; (c) SSS; (d) MLD; (e) EKE.

## Data Availability

The preprocessed acoustic data, environmental data, and associated codes are available from https://anonymous.4open.science/r/SSL‐CF12.
