# Supplementary material for: The Spatiotemporal Patterns and Environmental Effects of Pelagic Communities in the Northwest Pacific Ocean Based on Sound Scattering Layer Descriptors
Source: Ecol Evol. 2026 Jul 12;16(7):e73947. doi: 10.1002/ece3.73947 (PMC13357701; doi:10.1002/ece3.73947)
Supplement: Supplementary file 1 — Figure SA1: SSL descriptors used in this study. Figure SA2: Distribution of environmental variables in the optimal GAM for 2022. (a) SSH; (b) SST; (c) SSS; (d) NPP; (e) MLD. Figure SA3: Distribution of environmental variables in the optimal GAM for 2023. (a) SSH; (b) SST; (c) SSS; (d) NPP; (e) EKE. Figure SA4: Distribution of environmental variables in the optimal GAM for 2024. (a) SSH; (b) SST; (c) SSS; (d) MLD; (e) EKE. [file ECE3-16-e73947-s001.docx]

**Appendices**


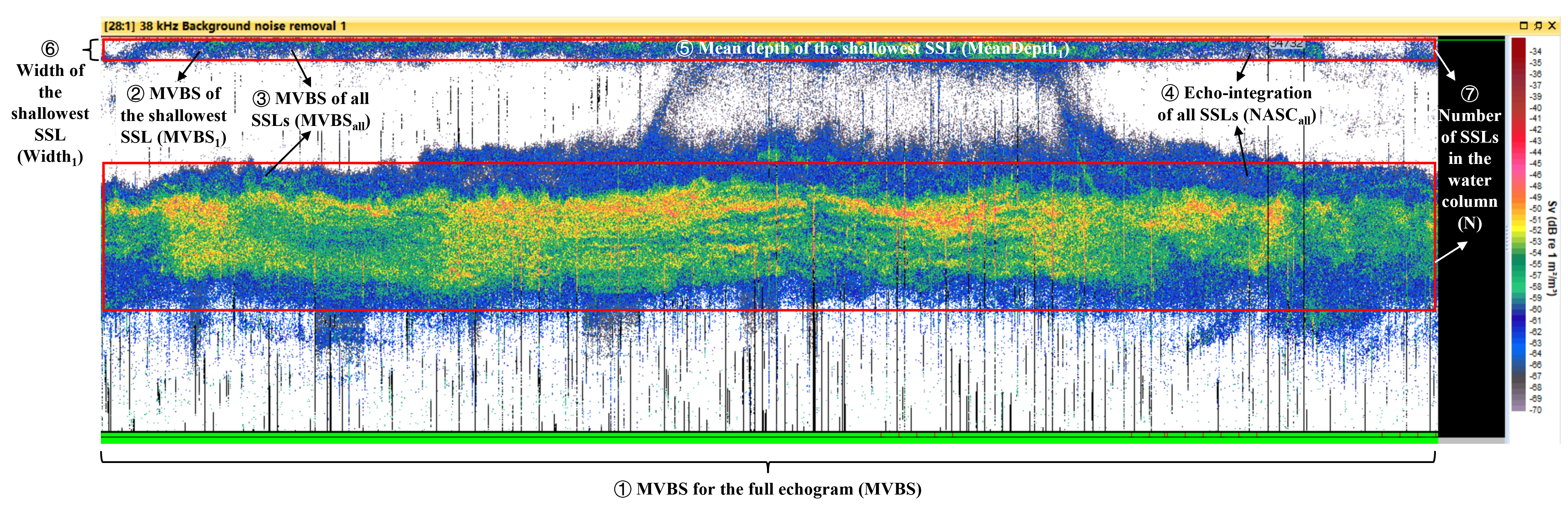


Fig. A1. SSL descriptors used in this study.


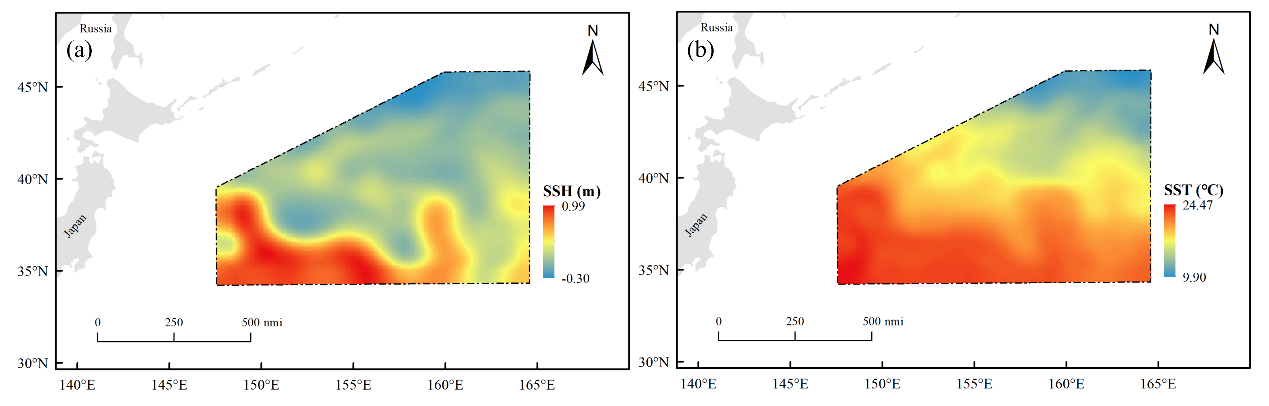


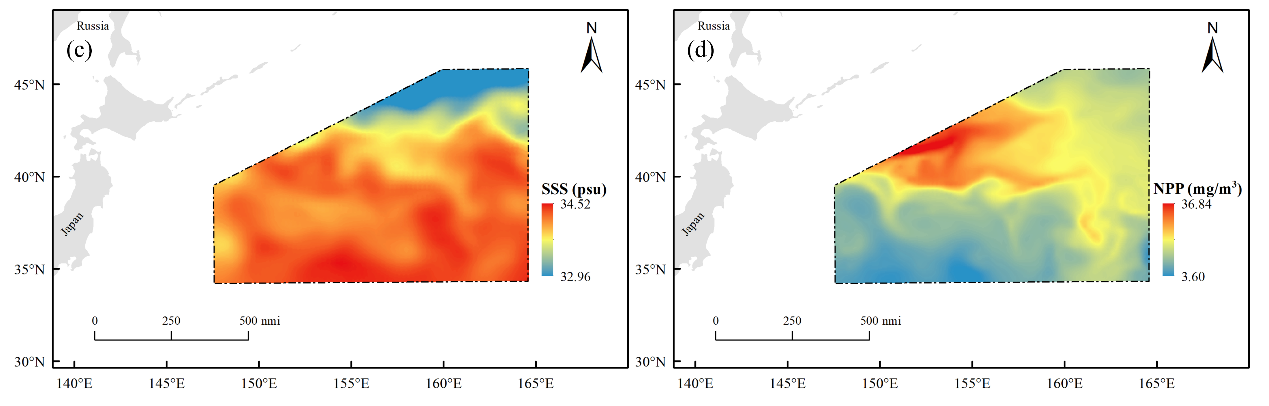


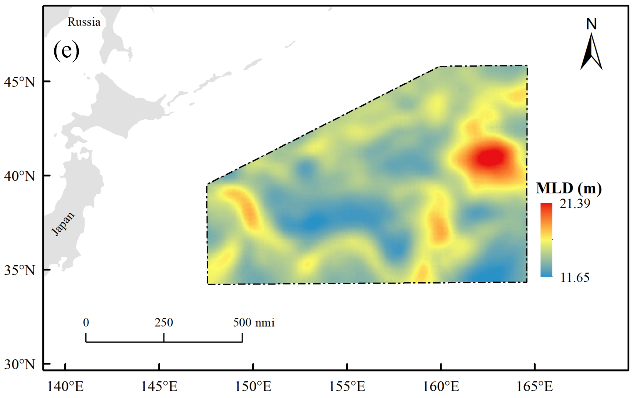


Fig. A2. Distribution of environmental variables in the optimal GAM for 2022. (a) SSH; (b) SST; (c) SSS; (d) NPP; (e) MLD.


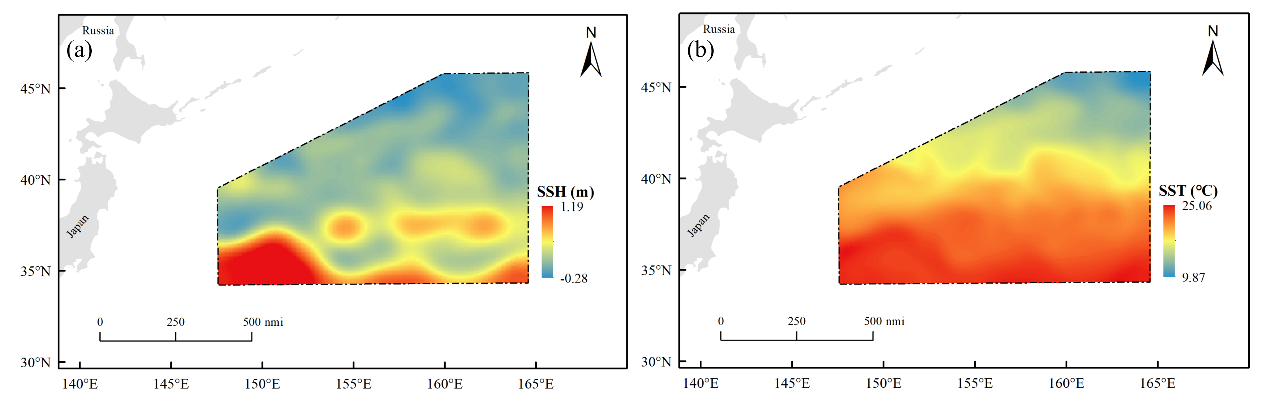


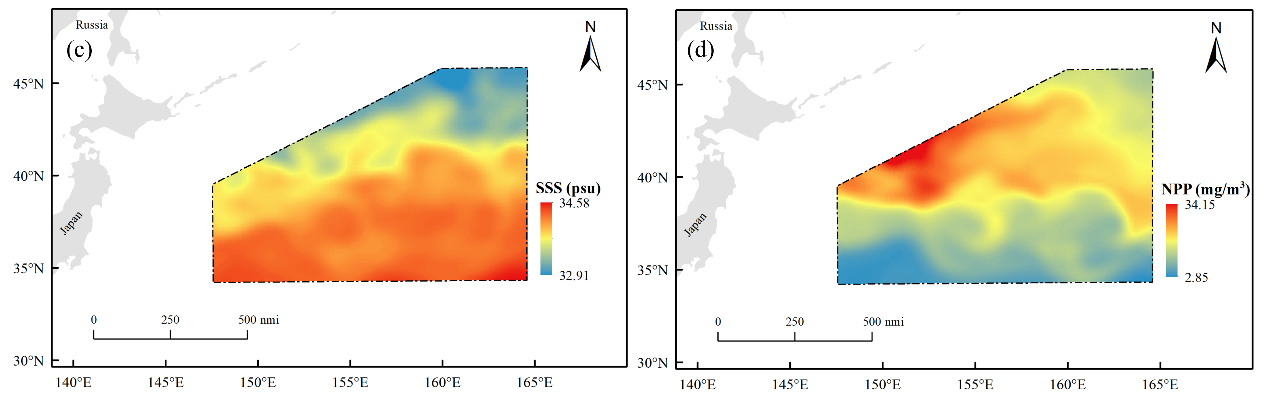


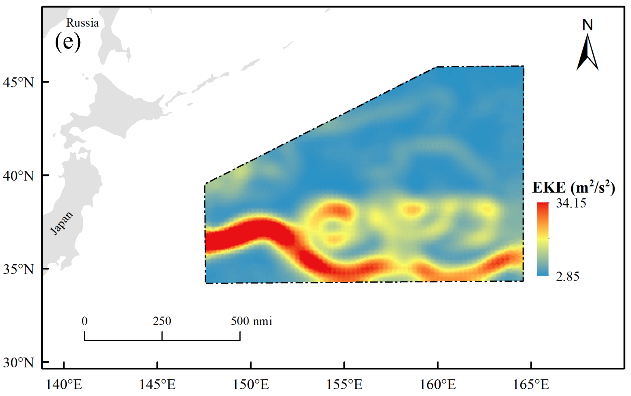


Fig. A3. Distribution of environmental variables in the optimal GAM for 2023. (a) SSH; (b) SST; (c) SSS; (d) NPP; (e) EKE.


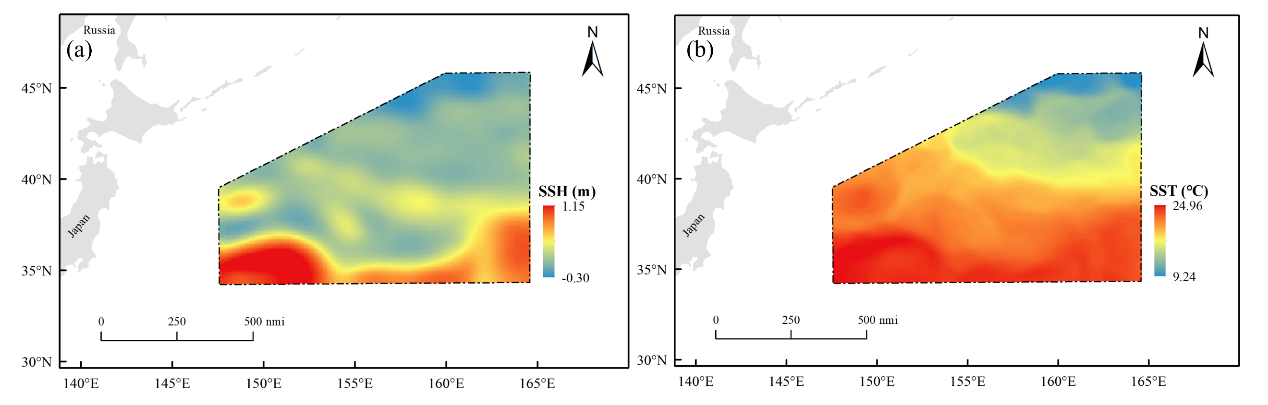


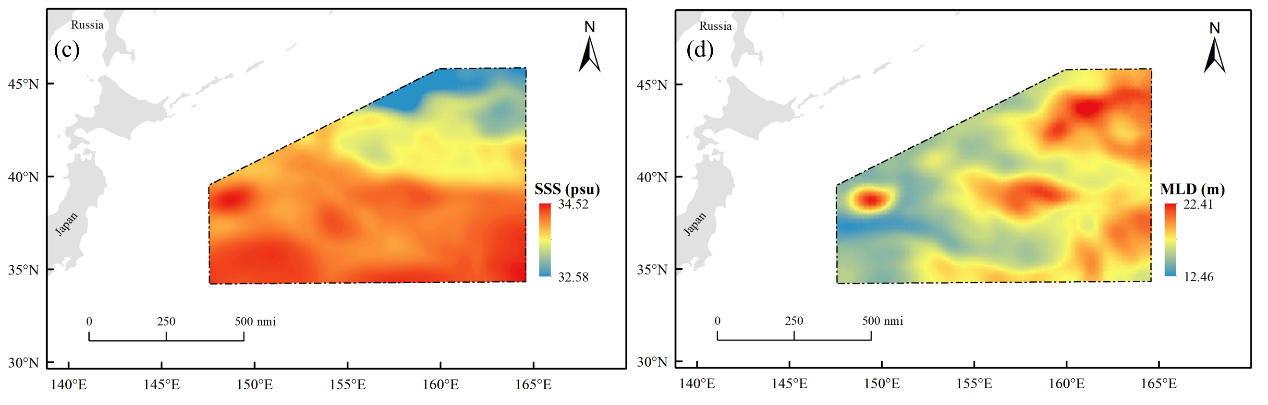


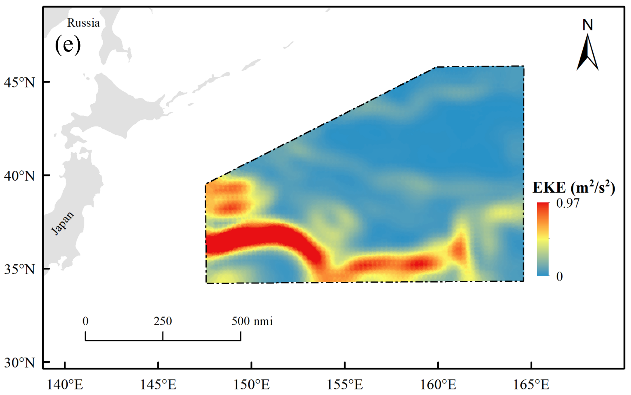


Fig. A4. Distribution of environmental variables in the optimal GAM for 2024. (a) SSH; (b) SST; (c) SSS; (d) MLD; (e) EKE.
